# A technology transfer journey to a model-driven access control system

**DOI:** 10.1007/s10009-023-00697-z

**Published:** 2023-02-10

**Authors:** Martina De Sanctis, Amleto Di Salle, Ludovico Iovino, Maria Teresa Rossi

**Affiliations:** 1 Computer Science Scientific Area, Gran Sasso Science Institute, L’Aquila, 67100 Italy; 2 Human Science Department, European University of Rome, Rome, 00163 Italy

**Keywords:** Access control system (ACS), MDE, Near-field communication (NFC), IoT

## Abstract

In the model-driven security domain, access control systems provide an application for handling access of persons through controlled gates. A gate, such as a door, can have a lock mechanism for securing the area from unauthorized access. Most commercial solutions for access control management offer pre-packaged software systems where customization of the authorization logic is either not allowed or subject to payment. Moreover, cross-platform development is a barrier for solution providers due to the high cost of development and maintenance that it implies. To overcome these limitations and further optimize the entire access control systems development process, we propose a model-driven approach that supports automatic code generation to enable communication between an IoT infrastructure and platforms for Facility Access Management. Specifically, the approach combines the benefits of Near-Field Communication (NFC) and Tinkerforge (i.e., an open-source hardware platform) with model-driven techniques. This allows the approach to exploit both behavioral and structural models for the modeling and the consequent code generation of part of the authorization mechanism, thus providing complete coverage of the code generated for the whole system. We implemented and evaluated our approach in a real-world case study within the premises of a fitness center with an IoT infrastructure consisting of several heterogeneous sensors by showing its practical applicability. Experimental results demonstrate the effectiveness of our approach in supporting abstraction and automation concerning traditional code-centric development through code generation features. Consequently, our approach makes the whole development process less time-consuming and error-prone, thus reducing the system’s time to market.

## Introduction

An Access Control System (ACS) provides a security application to check entries via controlled gates to restricted access areas [1]. The gate, such as a door, can have a lock mechanism for securing the area from unauthorized access. Then, the door is connected with one or more sensors triggering the control mechanism through communication with the rest of the infrastructure. In particular, access control is one of the security concerns that are addressed by model-driven security (MDS) approaches, which emerged for supporting the development of security-critical systems [2, 3]. In this domain, the Internet of Things (IoT) plays a central role since it refers to integrated networks of interacting devices.

In traditional chip card-based ACS, the standard plastic card containing an embedded microchip has to be physically inserted in a reader to be read; in case data stored on the chip allows access, it enables the electric door lock. Usually, the card reader hosts the logic for the access control, and for each equipped door, the code implementing this logic has to be distributed and released to the reader(s). In addition, a human resource management system is used to associate a person with each card, thus having a registry to manage the loss of cards or required checks.

In order to migrate an old chip card-based ACS to modern technologies supporting people authorization and authentication, a promising solution might be provided by NFC technology [4]. It enables the “touching paradigm” where the interaction can be identified as “the deliberate bringing together of two devices, for the purpose of obtaining services” [5]. Specifically, NFC requires two compatible devices to be close to each other to enable contact-less identification and interaction. Moreover, most commercial solutions for access control management also offer pre-packaged software systems for human resources management. The main issue with these existing solutions is the impossibility of customizing the authorization logic provided by the tool vendor. The authorization logic may reside on the NFC reader or a remote server, communicating with the devices depending on the adopted architecture. However, if customization is provided, it comes as a fee-based service, often quite expensive.

To overcome this limitation, we may benefit from technology such as Tinkerforge. Tinkerforge [6] is an affordable open-source system of building blocks using the concept of a pluggable module, helping to simplify the implementation of IoT-based systems. The implementation of the building blocks is based on intuitive API bindings available for many programming languages, such as Java, PHP, and Python. This system, offering a high degree of abstraction, might ease the technical implementation with less code to be developed concerning other components. The main advantage of selecting a target technology such as Tinkerforge is that it can be used to build systems interacting with other platforms and customize the access control algorithm for the required purpose since the code has to be developed. Moreover, the building blocks of the infrastructure can be selected and mounted based on the requirements and easily extended. Although this selection offers a good trade-off between development and provided functionalities, the code controlling the devices must be manually written. However, by combining the benefits of NFC and Tinkerforge technologies with model-driven techniques, *code generation* may be applied to reduce the burden of manually developing the code for the ACS.

Actually, model-driven engineering (MDE) [7] aims at tackling the complexity of software systems via abstraction, using models as first-class entities. Models are employed both for descriptive and prescriptive purposes [8]. Model-driven development (MDD) approaches open to code-generation strategies to reduce time to market and improve software quality [9]. Furthermore, the model-driven architecture (MDA) paradigm provides support for the definition of model-based system structure with the so-called Platform-Independent Models (PIM) and Platform-Specific Models (PSM). PSM describes the functionalities defined in the PIM for a specific implementation technology. Then, by defining transformation mapping, we can traverse different abstraction layers [10]. Indeed, *model transformations* can be successfully employed to support platform-independent to platform-specific translation and implement multi-platform code generation from a single input model of the system [11, 12]. This is quite relevant given that, nowadays, cross-platform development is a barrier for solution providers due to the high cost of development and maintenance of targeting development to different platforms. Lastly, one can state that (especially in the industry) proprietary solutions may be employed to automate the development and deployment of the needed code. To the best of our knowledge, ThingML [13] is the first attempt to create a model-driven framework with generative features for IoT systems. However, in many cases, a ThingML component has to communicate with external closed-source components, e.g., Tinkerforge technology, whose implementation cannot be modified. Since the only option is to extend and adapt ThingML through different extension points (see [13]), evaluating the effort for this kind of task is fundamental. Moreover, the trade-off for selecting these existing approaches relies on technical details shifted from models to code generators or moving them from PIM to PSM. For this reason, it could be worth implementing a new domain-specific language with dedicated support when technology offers a wide range of hardware and software components.

With these premises, we propose a model-driven approach for access control systems that supports automatic code generation to enable communication between an IoT infrastructure or an existing Web platform for Facility Access Management. The presented approach can generate software components in the selected target language. This way, it reduces the implementation effort by producing the code needed for the interaction while making the whole development process less error-prone and time-consuming. This paper extends the work presented in [14], where the approach was first introduced, in the following points:

- The positioning of the approach in the *Model-Driven Security* domain [2], and its definition according to conventional access control enforcement framework [15];
- The infrastructure metamodel, in order to support *additional sensors* in the code generation;
- The overall modeling approach to further automate the *code generation from behavioral models*, besides structural ones. This allows the approach to share a common set of behavioral and structural models satisfying the specific needs of ACS that can be further reused and customized, as in software product lines approaches [16], thus managing the variability of IoT devices and platforms;
- A new architecture including a repository of authorization policies that can be further extended by providing query scripts predicating on models;
- The *evaluation* of the approach through a *real-world case study*, by developing and deploying an ACS on the premises of a fitness center.

The paper is organized as follows: Sect. 2 explores the background and related works and discusses how and to which extent our approach aims to broaden the literature. Section 3 offers a real case study for introducing the domain in which the proposed approach has been validated. In Sect. 4, the approach is presented with all the involved model-based artifacts, whereas in Sect. 5 it is applied to the presented case study. An evaluation has been conducted and is discussed in Sect. 6, while Sect. 7 draws some conclusions and discusses future developments.

## Background and related work

This section revises (i) ACSs approaches to show their limitations and (ii) those techniques we use in this work (e.g., MDE, domain-specific language, automatic code generation) since they have already been used in contexts similar to ours, showing their effectiveness.

*Access Control Systems* provide a security application to check entries via controlled gates to restricted access areas. Their application is foreseen in both out-door [17, 18] and indoor [1] environments through diverse technologies. Moreno et al. [1] describe a distributed access control mechanism in a smart building scenario. An engine embedded into smart objects makes authorization decisions based on user location and access credentials, where user location data are estimated via magnetometer sensors in smartphones. The authors highlight the potential of the approach since no additional hardware or infrastructure is required. However, smartphones’ power consumption should be considered since it can hinder the functioning of the approach. Shao et al. [19] present a wireless and device-free door access system, called *RFDoorGuard*, which leverages received *signal strength indicator* (RSSI) signals from Bluetooth Low Energy beacons to recognize people entering a room. They evaluate RFDoorGuard in two real scenarios, an office room with the key lock and a meeting room with swipe card access. This work aims to verify whether users can be identified without using any devices or keys. However, the known accuracy limitations related to the chosen Bluetooth technology can lead to the malfunctioning of the system. A home security system based on human motion detection and remote monitoring technology has been developed by Anwar et al. [20]. Such a system provides features to confirm the visitor’s identity and control door accessibility. Human detection has been implemented through a *passive infrared* (PIR) motion sensor and a camera module. Instead, the door accessibility is deployed with an electromagnetic door lock module. The authors envisage the control of the system through the use of smartphones that, as highlighted by Moreno et al. [1], could lead to different limitations (e.g., battery level). Another home security system design based on human face recognition technology and remote monitoring technology is reported by Sahani et al. [21]. In this security system, door accessibility is implemented through the combination of a ZigBee module and an electromagnetic door lock module. In his work [22], Gossen proposed an approach for exploiting human face recognition in access control systems, to support people identification in a contact-less manner. Specifically, it relies on two known holistic methods for facial recognition (i.e., Eigenfaces and Local Binary Pattern Histograms), and it enriches them with precise alignment of face images. Despite the use of low-cost technologies, mere human face recognition could affect the proper functioning of the approach in terms of trustworthiness.

*Model-Driven Engineering* (MDE) has already been adopted to deal with the challenges of the IoT domain (e.g., devices’ heterogeneity, reusability of software artifacts) [23]. Prehofer et al. [24] present two different approaches for the development of an IoT application, namely IoT Mashups and Model-based IoT. The first one uses existing services/tools to develop applications, while the second one exploits MDE techniques. The authors compare the two approaches and show that mashup tools efficiently describe system architectures, message flow, and deployment. In contrast, model-based approaches have a greater expressiveness in modeling different points of view and behaviors and generating code from models for different platforms. Also, Amrani et al. [25] propose a high-level rule-based language to model high-level representations of devices to manage the interoperability and behavior of interconnected IoT devices.

*(Semi)-Automatic code generation* for architectural models has gained much attention in recent years, even in the IoT domain. Template-based code generation (TBCG) emerged as an approach to develop code generators [26]. Different templates can be used as inputs for automatic code generation, defined with diverse modeling languages, e.g., a domain-specific language (DSL) or a general-purpose language, like UML. As regards specific UML-based code generation, Pham et al. [27] exploit MDE to generate executable code from state machines. In particular, the presented approach provides a pattern and tool for code generation from UML State Machine, extending IF-ELSE-SWITCH constructions of programming languages with concurrency support. They show that with their approach, the UML semantic conformance is respected. On the contrary, Sunitha et al. [28] highlight the fact that in the implementation of automatic code generation from state diagrams, a significant obstacle relies on the absence of a one-to-one correspondence between the elements in the statechart diagram and the programming constructs. Thus, the existing programming elements cannot implement two main components related to the state diagram, namely state hierarchy, and concurrency. The authors present a design pattern for implementing the state diagram, which includes hierarchical, concurrent, and history states. If we look to domain-specific approaches instead, Marah et al. [29] present a Domain-specific Modeling Language for TinyOS, called *DSML4TinyOS*, whose aim is that of allowing developers to generate architectural code for low power wireless devices. DSML4TinyOS language aims to provide a platform-independent modeling framework to abstract from different IoT operating systems by exploiting model-to-model transformation and code generation. Nguyen et al. [30] presented *FRASAD*, a framework providing a fast way to develop IoT applications using model-to-model transformation and code generation, running on top of a software architecture based on the sensor node domain concept. They aim to improve reusability, flexibility, and maintainability in the IoT applications development by proposing a multi-layered software architecture leveraging a high abstraction level. We share the same objectives with [29, 30], in the proper ACS context, with its specific needs (e.g., authorization).

*Low-Code Platforms* have been introduced in the IoT domain to deal with the high heterogeneity of devices and architectures [31]. Ihirwe et al. [32] extract the functionalities and limitations of existing approaches supporting low-code development platforms in the IoT domain. The authors noticed the lack of support for testing and analysis in the IoT systems development process and standards to support the model-based development of IoT systems, which compromises interoperability among different platforms. It also emerges the limited use of multi-view modeling. In particular, the authors state that the existing approaches use one specific view to model everything. The study results also show limited support for cloud-based model-driven engineering since most existing tools still require local deployments. Also, Sanchis et al. [33] highlight the lack of transversal platforms developed on open standards to create ecosystems to automatize the IoT systems development. Moreover, they extracted some key challenges of low-key platform development. They are related to real-time data processing issues and the integration of secure code analysis into the development workflows. However, the authors highlight the importance of low-code development platforms that may represent the response to new digital companies’ requirements for faster automation software development.

*Domain-Specific Languages* (DSLs) have been adopted mainly to software development in different contexts [34]. For instance, in the context of Web applications development, a DSL for the implementation of dynamic web applications was presented by Groenewegen et al. [35] and further extended in [36]. To enable user-level software development and rapid prototyping of sophisticated web applications, Boßelmann et al. [37] introduced a framework called DIME, which supports the Extreme Model-Driven Design (EMDD), by providing a family of graphical domain-specific languages (GDSLs) each of which covers a specific aspect of modern web applications, including data model, business logic, user interface, access control. In the context of access control, we have examples of approaches using DSL specifications. In particular, Ben Fadhel et al. [38] proposed a DSL whose semantics can be expressed in terms of a formalization of rule-based access control policies as Object Constraint Language (OCL) constraints on the corresponding conceptual model. This approach aims to define more expressive and clear policies and implement a method that allows the easy reinforcement of these policies. Moreover, Salman et al. [39] introduced a DSL called *SenNet* for developing Wireless Sensor Network (WSN) applications. It has been implemented to reduce the complexity of low-level programming details associated with the domain of WSN. A further application of DSL in the IoT context to reduce its inherent complexity has been presented by Salihbegovic et al. [40]. Their work presents an editor, called *DSL-4-IoT*, implemented to deal with the high complexity and heterogeneity of WSNs and devices. The editor outputs IoT application configuration files which are then executed by a runtime engine (i.e., OpenHAB). However, we aim to go beyond by combining the benefits of NFC and Tinkerforge technologies with model-driven techniques, thus bringing their advantages altogether.

In summary, the literature highlights that the development of ACSs has gained attention in the last years, due to their relevance and effectiveness, especially in certain contexts where security requirements make them quite essential. As reported above, different approaches have been proposed by exploiting diverse technologies and methodologies applied to the IoT domain. In this work, instead, we aim to exploit the before-mentioned benefits of MDE, DSL, and code generation-based approaches by also combining them with the use of the Tinkerforge platform. This combination allows our approach to significantly push and increase *modularization*, *abstraction*, and *code generation* features (as detailed in Sect. 4 and demonstrated in Sect. 5). The application of the presented approach in a real-world case study further shows the reduction of implementation efforts and time to market (details in Sect. 6), which might favor the realization of more reliable solutions. Moreover, the open-source nature of the Tinkerforge platform further allows us to overcome the rigidity of commercial solutions for access control management in terms of *customization* features. Eventually, the provided infrastructure is *open* and *extensible* to different technologies and device types besides RFID/NFC.

## Case study

**Fig. 1:**
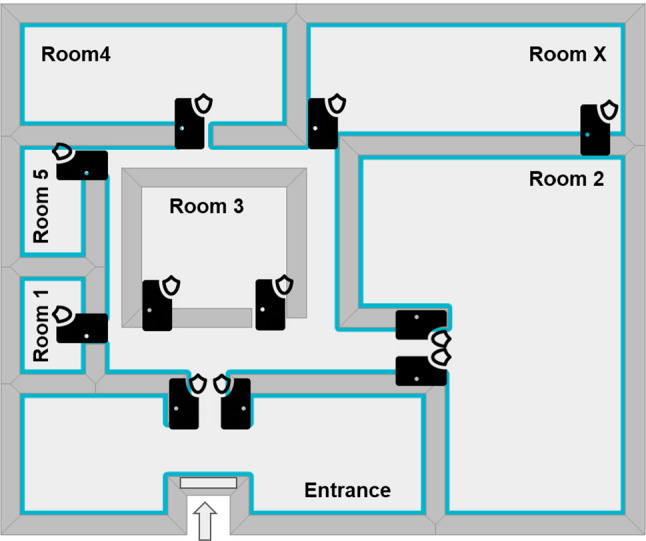
Layout of the building

In this section, we expose the case study we designed from a project experience with a company^1^. Specifically, the company we deal with is a fitness/physiotherapy center that is organized in diverse rooms, as represented in the layout in Fig. 1, distributed in a building of 1600 square meters. Each of the seven rooms is equipped with different facilities to host-specific activities (e.g., training, rehab, pilates, dance). Activities are scheduled in different time slots from Monday to Saturday, with various frequencies, and assigned to the available rooms. The clients of the center can enroll in different courses by choosing different subscription plans and offers.

The company wants to exploit the subscriptions that clients hold to automatically check if they have access or not to each specific room in the building. This way, it can be easily verified if only authorized clients, those regularly registered in certain activities, have adequate access. Every room can show different requirements from the IoT-based infrastructure perspective. For instance, the main entrance is subject to different needs and environmental requirements with respect to other rooms like *Room 2*, where activities are performed. Some of the rooms, i.e., the biggest ones, can be equipped with two doors or gates.

The authorization and identification of a user (client) are then delegated to the access control infrastructure that, in this case, is implemented with the selected NFC technology. This technology is essentially made by an NFC Reader mounted near the door of a room that will identify the NFC tag used by the client (usually integrated into a bracelet) and, in turn, trigger the authorization mechanism by also communicating with the ACS, which can be deployed in the cloud (i.e., external deployment) or the intranet (i.e., internal deployment). For instance, this can happen through the interaction with Web Services or APIs devoted to this task and exposed by the ACS. In our case study, we make use of an API already in use by the fitness center, exposed by a Web system managing the clients and subscriptions. The devices controlling the door locks invoke the REST API, and in the case in which the user is unauthorized, it will return an empty set. The JSON response of the authorization REST API will be read by the device that will grant/deny access to the room.

Tinkerforge is an open-source platform of building blocks belonging to three categories, namely *Bricks* that can control different modules called *Bricklets*. Then, the communication interface of these building blocks can be extended by using *Master Extensions*. More precisely, each brick has one task, which in our case study consists of checking the communication and authorization. Bricks are stackable in the sense that they can be mounted on top of each other by creating a hierarchy. For simplicity, in this paper, we consider non-stackable bricks. Bricklets can be connected to a brick with a cable, and depending on the bricklet type, different sensors, displays, I/O interfaces can be added. Master extensions are, instead, used to change the interface from USB to Ethernet or Wi-Fi.

**Fig. 2:**
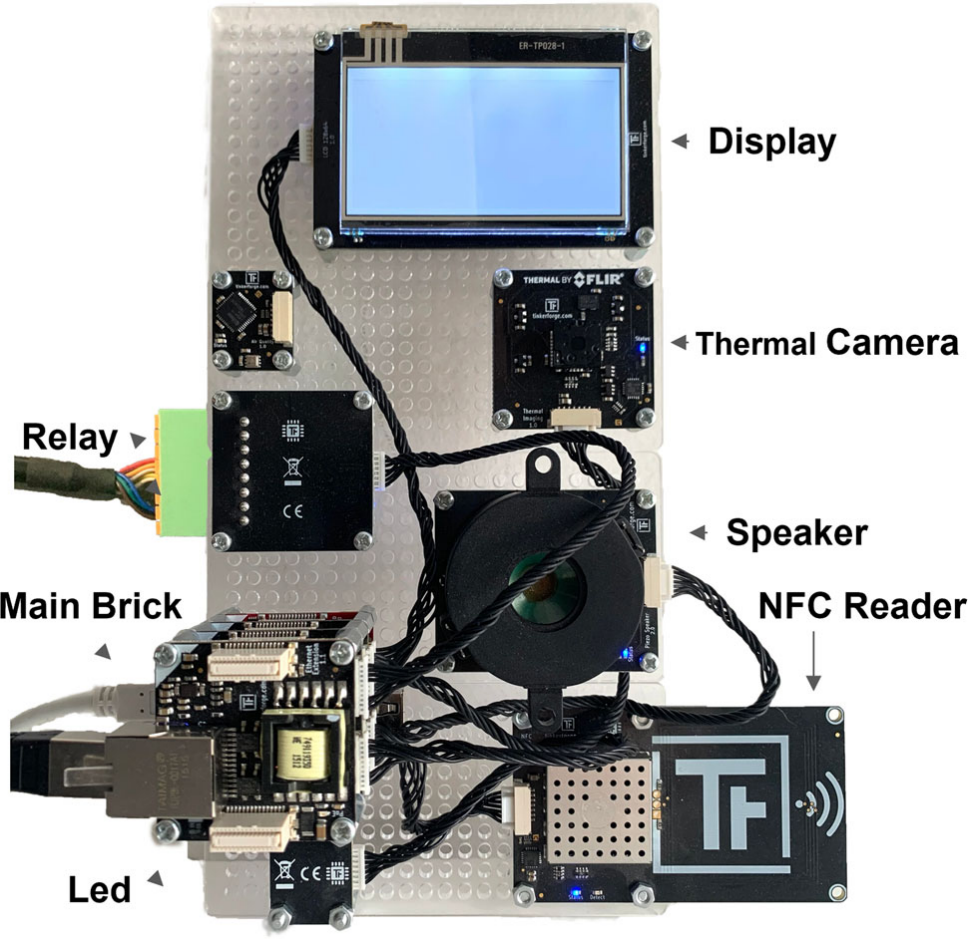
Picture of the assembled device

To support a basic configuration of the ACS, we can consider an architecture composed of a single brick per door (of a room) and multiple connected bricklets (as depicted in Fig. 2). Depending on the needs, bricklets can be an NFC reader, a speaker (beeper), a led display, or a relay to control the door lock. Relays are switches that can electronically open and close circuits. In our case study, they control four circuits (that is why this component is also called a quad relay). Moreover, each client has to receive an NFC tag (i.e., a card, sticker or bracelet) to be used for entering the fitness center premises, if authorized. To this aim, NFC tags must be enabled according to the profile of their owners. Enabling a tag means adding it to the ACS software that manages the users and their subscriptions. The main advantage when autonomously designing the hardware configuration of each door gate is that we can address different requirements and needs so that every door can be equipped with different devices. For instance, since the entrance is the bottleneck of the building, where every person should pass in order to reach interior rooms, the speaker could not be mounted to reduce the noise in that area. Another option could be that the interior rooms are not equipped with a display for showing the information about memberships or the next subscription deadlines since the main gate is located at the entrance, and this information can be displayed only once. This mechanism also permits to reduce the hardware cost by equipping the building with what is needed, and not a pre-packaged solution. This technology basically allows for two possible architectural deployment schemas: (i) deploy the developed code communicating with the bricklets directly on board using a proprietary deployer tool, i.e., the entire device equipped with an additional specific brick called *red brick*; (ii) deploy the whole code on a client or server machine that will establish a connection through an IP address with each brick, and then communicate with the corresponding bricklets transparently. Both these options are valid; in this work, we decided to test the centralized architecture with a server. Traditional code-centric techniques, in this case, would be costly since the same developed code cannot be deployed on every device due to the before mentioned variability.

Figure 2 shows the prototype complete of all the bricklets used for testing our approach. In particular, we can spot the main *Brick* with the connected PoE (Power Over Ethernet) cable, the *Led* that can be used to blink in different colors (e.g., green or red), the Oled *Display*, the *Speaker*, the *NFC Reader*, the *Relay* used to open or close the electric circuit of the door lock. In particular, the *NFC Reader* is mounted in such a way to be out of the case of the board. Eventually, a *Thermal Camera* has been mounted to make the example even more complex and to offer a temperature scanning feature due to the Covid-19 restrictions of this period. This is the full configuration of the device, i.e., the one used in the entrance room.

Typically, conventional access control enforcement frameworks [15] include a Policy Enforcement Point (PEP), a Policy Decision Point (PDP), a Policy Administration Point (PAP), and optionally a Policy Information Point (PIP). Briefly, the user requests access through the PEP, which will forward the request to the PDP and evaluate the access request against the authorization policy. Usually, at this stage, the PDP refers to a policies repository, e.g., a database query, and when the evaluation is complete, it returns the decision to the PEP. The PEP decides to grant or deny access to the user for the specified resource, i.e., in this case, a room, and the authorization request can be enriched with additional information by the PIP, e.g., user rights. The last component is the PAP, which is responsible for administrating the authorization policies.

In this case study, the basic steps to be performed when entering a room are the following: the NFC reader, embodying the PEP, receives the NFC tag identifier (from the client), and it checks the authorization with the ACS, asking the PDP. If the authorization is granted, the board emits a beep by using the speaker. Then, it shows an authorization message on display, and the led can blink green. In the end, it enables one of the channels of the relay to control the door lock. An additional check can be conducted by measuring the body temperature using the thermal camera. If the authorization is denied, instead, the NFC reader emits a different sound and displays the error message, and the led blinks in red. All these operations can be implemented using the Tinkerforge API to interact with the board and all the equipped sensors from the server. We outline that the code, both when used in a centralized architecture or deployed on the brick, acts basically in the same way.

In the following, we show the Java code that must be manually written, in the absence of automatic code generation, to implement the explained control logic among the various sensors and bricklets, for our case study, as explained in the previous paragraph. Listing 1 reports the Java code developed for the logic of a single brick (the one previously presented and depicted in Fig. 2). This initialized brick has a UID (identifier) as all its bricklets and will be powered by PoE extension, with IP address localhost in the communication LAN.
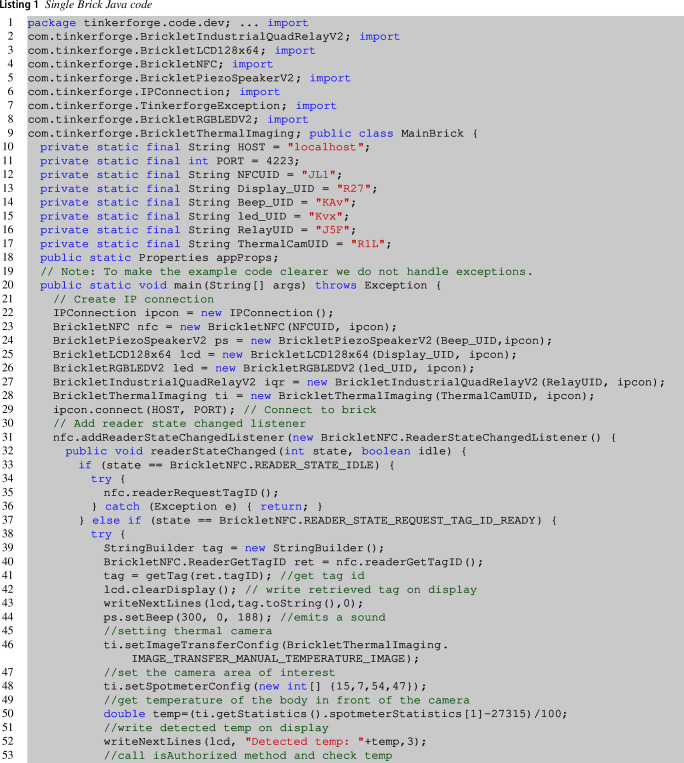

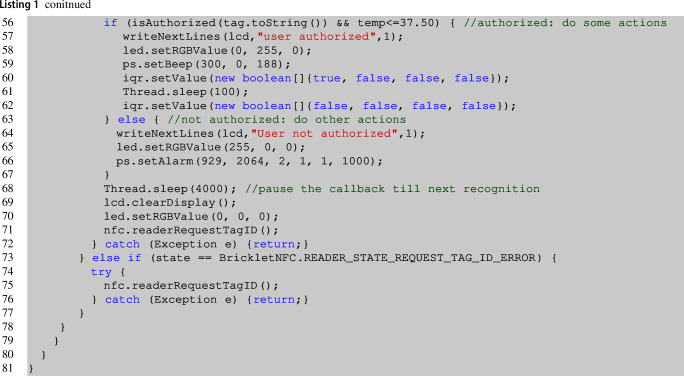


Since this brick has different types of connected bricklets, e.g., NFC Reader, Relay, etc., we have multiple variables points in this code. First, lines **3–10** include selective import statements for the API bindings needed to the main brick to interact with the mounted bricklets. Then, lines **12–19** declare the variables needed for initializing these modules. Every bricklet has a UID that has to be specified in order to be able to interact with it. Then, in the main method of the brick in line  **31**, the connection is established. Lines  **25–30** initialize all bricklets part of the chosen architecture using Java classes included in the developed project. From line  **34**, the NFC reader declares its states. In particular, when the reader is in the IDLE state, it requests a tag until the tag is found (line  **42**). From this line, the core code begins. Lines  **43–54** retrieve the tag id, and a series of actions are undertaken to interact with the bricklets. Namely, the display is cleared, and the new tag retrieved is printed on display. A sound is emitted from the speaker, and the body temperature is obtained from the thermal camera. This first set of actions is part of another variability point that is more evident from line  **56** to line  **67**. In fact, in these lines, the authorization mechanism is executed. At line **56**, the method isAuthorized is invoked, and the temperature is also checked in the positive case. This method simply calls the REST API with the detected tag as a parameter, and it returns true or false for the granted or denied authorization, respectively.

Lines 57–59 report the sequence of actions where all the bricklets are called by the brick, e.g., it writes a string on display to make clear that the user is authorized, the led becomes green, it emits a specific beep, and interacts with the QuadRelay component, to open and close the selected channel. This method takes as input the channel’s position to open and blink for the selected time-lapse. Then, it opens the relay and further closes it (lines 60–62).

Lines 64–66 execute the brick’s actions in case the user is not authorized. It writes on display, blinks with the led in red, and emits an alarm sound. This code must be deployed on the device (another brick is needed, but we leave out of the discussion this aspect) or on a server starting the threads using the devices’ IP address.

Eventually, there is also the need for code to start a thread for each brick in the infrastructure. In our case study, we considered 11 bricks (as much as the room doors), but we just reported the code for the one mounted at the entrance in the previous listing. We outline again that each brick can be configured with different bricklets, depending on the architect’s decisions and specific needs. More specifically, one brick can be equipped with a display and another one with a speaker, depending on the area’s noise level. It will make the initialization code different from one brick to another. To better explain the approach through the real case study, we refer again to the planimetry of the building shown in Fig. 1.

Entrance This room is equipped with the brick and all the bricklets, as reported in Fig. 2, comprising the thermal camera. It prevents the client from entering the building, thus avoiding entry to the rest of the rooms if access is not granted. All the bricklets can be needed, and, due to Covid-19 restrictions, those subjects whose body temperature is higher than 37.5$^\circ$C should not be allowed to enter the facility. We outline that this check could be performed at the entrance and not repeated at every gate, allowing us to save the equipment budget. For this reason, a thermal imaging bricklet is mounted only at the entrance in order to support this feature.

Rooms 1–5 These rooms are equipped with bricks and all the bricklets as reported in Fig. 2, with the exception of the thermal camera and possibly of the display that would be redundant in these rooms.

Room X This room is equipped with a brick and all the described bricklets except for the thermal camera, led, and display. This choice is guided by the room’s environmental requirements since it hosts a swimming pool. Thus the device is covered by a plastic case to avoid water contact.

This case study configuration highlights that the various devices are configured with different sensors and bricklets. The code to interact with the devices can be different depending on the location of the subject device. In this simple scenario, the *variability* is quite evident, but we could have more complex scenarios. Specifically, we have all the devices with different codes since every mounted bricklet and each brick has different identifiers and configurations. There are also three different modular configurations for the three scenarios (i.e., room types) of the building. Variability is often managed with modeling techniques, such as modeling feature models and software product lines. The variability in modeling is defined as the most advanced approach to define the commonalities and variabilities of reusable artifacts such as software components [16, 41].

**Table 1:** Variability for single brick code

Code Lines	Code	Dependence
3–10	Selective import statements	Structural
12–19	Selective variables declaration	Structural
25–30	Selective object instantiation	Structural
57–66	Bricklets interactions	Behavioral

Table 1 summarizes the brick’s variability aspect whose code is reported in listing 1. The first column details the lines of code affected by this variability; the second reports the type of code involved, and the third the type of dependency. The type of dependency identified in the presented case study can be *structural* or *behavioral*—the former highlights that the lines of code can vary with respect to the brick’s chosen architecture. At the same time, the latter refers to the control logic among the various bricklets. The first row specifies the import statements related to the mounted bricklets. The second and third rows of the table are about objects declaration and instantiation, strictly related to the mounted bricklets. Thus they also refer to a structural dependency. Those lines of code induce the possibility of using the bricklets or not. A misconfigured application can lead to errors when using bricklets that are not present in the current architecture. The last row is about how the developer wants to drive the interaction between the mounted bricklets, and for this reason, it refers to a behavioral aspect of the application.

Due to this variability, manual coding can be error-prone, time-consuming, and requires attention to customize the configuration of the case study and the real-world application. Programmers are used to applying copy-and-paste commands since the software managing the devices will be similar. However, some parts are custom, inducing errors in the case of code clones [42] and variable declarations. A typical example of an error introduced in this scenario is a wrong bricklet UID (as a result of copy and paste operations) attempting to connect to a device that is mounted on another brick. Moreover, software maintenance in this type of application is quite difficult since the extension of the architecture or later modifications can lead to quality degradation. The more the lines of code increase, the more the number of possible introduced errors can rise.

## A model-driven ACS

**Fig. 3:**
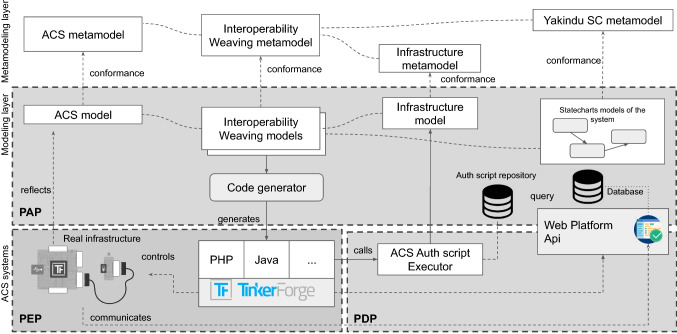
General overview of the approach

**Fig. 4:**
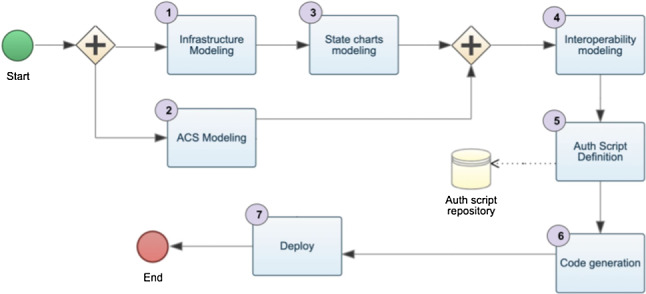
Overview of the process

This section provides a general overview of the proposed approach, which empowers the developers to realize access control systems without considering the implementation details. Figure 3 reports an abstract representation of the architecture of our approach, mapped on the conventional access control enforcement framework’s structure. Figure 4 shapes the process execution labeled with .

An ACS is composed of multiple hardware devices and running software systems (bottom-side of Fig. 3) composed of real infrastructure, together with the code managing all the devices that can be deployed on a server or deployed on each device (see Sect. 3 for an example). The system, running on the device, reacts at the user request activated by a tag touching the NFC receiver. This device implements the PEP of the framework, and it interacts with an *ACS authentication script executor*. The ACS script executor embodies the PDP of the framework since it is in charge of evaluating access requests against authorization policies before issuing access decisions. Indeed the executor invokes an authentication script, selected by considering the authentication policy stored in a *Auth script repository* that will be part of the PAP of the framework. The repository stores multiple authorization policies in the form of queries that the administrator can select, e.g., allowing access to users with memberships of a selected activity that runs on selected days, excluding public holidays, etc. The device implementing the PEP also communicates with the door lock to grant (or deny) access in response to the PDP decision. Basically, the device (PEP) sends the activated request by the user to the ACS Auth script executor (PDP) that is in charge of executing the selected query returning the authorization response to the device that will open the door or reject the request. The PDP may alternatively communicate with a Web platform requesting authorization for the user.

Possibly, a Web platform can be used to manage the clients/users of the ACS so that the system can be integrated with third-party applications. This has been placed between the PDP and PAP since the web platform can be used to request authorization (PDP) by REST call API but can also be used to manage the users and permissions, making it part of the PAP. The modeling layer (center of Fig. 3) made of software systems and hardware can be designed with the right level of abstraction and reflects what will be in the real-world system in the bottom layer.

The *Infrastructure* of every device mounted in the building can be represented with a model (modeled in activity  in Fig. 4), indicating how every device is assembled, with which bricklets in this case. This is particularly close to what in literature is called hardware description languages VHDL [43], used to describe the structure and behavior of electronic circuits. The *ACS* is then represented as a model (composed in activity  in Fig. 4), with concepts and relations needed to design the system and the building to be equipped. Within the ACS, the defined concepts permit to model elements such as rooms, clients, and activities. The system’s behavior can be represented in terms of events and flow of information embodied in their statecharts models (in activity , which is subsequent to activity ). The behavior allows guiding the interaction between the bricklets, the software system, and the user exposing the tag. A modeling project can be generated from the infrastructure model with a minimal statechart specification for every brick declared in the infrastructure, in . In this phase, the modeler will elaborate on the statecharts representing the brick behavior and consider the infrastructure specified in the model. In activity  we use weaving models [44] to compose *Interoperability Weaving models* that represent the following relations:

- The relation specifying where every device is mounted;
- The behavior associated with a specific brick.

This modeling layer reflects what the real-world system is and can be used as a prescriptive or descriptive model [45]. Activity  is in charge of defining authentication scripts that will be available through a repository and implementing authorization policies. This activity corresponds to what we would call defining queries but is executed on the models defined in the previous activities. Activity  generates the code by using the models defined in the previous activities that can be deployed on the devices in activity . In the rest of this section, we show how we have implemented the metamodeling layer (top-side of Fig. 3), allowing us to specify the models cited above. Our approach is fully model-based, and for this reason, we provide a query-based component for the authorization mechanism that has been replaced by REST API calls in the real system that we deployed in the fitness center. To this end, we show all the metamodels that engineer the concepts and relationships of the entities cited before. Eventually, we show how these models can generate the complete code for the ACS controlling the devices. This activity is triggered in  and finalized in . The activity in  might be continuously performed until the ACS modeling provides a stable and desired ACS strategy. The reasons to use PAP as a third-party web-based solution are multiple: first, the fitness center, used as a benchmark already, had an existing client management platform; second, the entire model-based solution would force the administrators to use modeling editors and Eclipse Modeling Framework (EMF)^2^ technology to manage also the clients, that would result limiting in terms of user interface for modeling editors and time-consuming for the needed training activities.

### Modeling ACSs

**Fig. 5:**
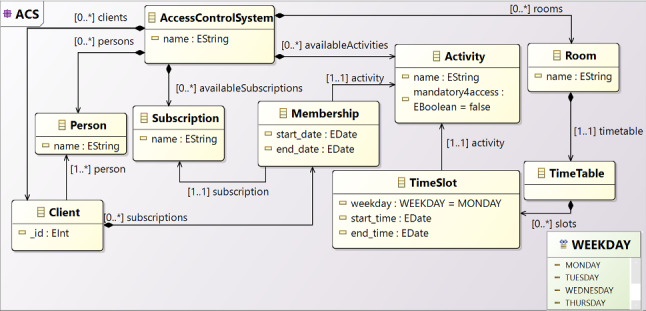
Access Control System Metamodel

We use a metamodeling approach to generally represent case studies like the one described in Sect. 3. To better comprehend the application domain, we engineered the requirements for ACSs in the metamodel in Fig. 5 (the ACS main package is reported). An *AccessControlSystem* is organized to manage a list of *Activities* that can be conducted in the facility and specifically in the available *Room*s in specific *TimeSlot*s. Each room has a *TimeTable* of activities, performed in specific *WEEKDAY*s and time. The timeslot can define exclusion criteria by declaring *toexclude* dates, so the regular schedule can be easily modified for a single date, e.g., excluding public holidays. The system can register *Client*s associated with a *Person* with all the fields for the identification (hidden for simplicity).

Each client can be linked to *Membership*s with a specific *start* and *end* date related to an *Activity*. The ACS can manage who is authorized to access a room on a specific date and time. This can be derived by retrieving all the client’s activities and matching them with the timetable of the activities. This metamodeling approach is flexible enough to represent a working facility where employees can be associated with the activities, i.e., job positions. Every position can have a deadline, i.e., the end of the contract. An office can grant access if the job position is associated with the room, i.e., an office or a meeting room, and the contract deadline is still active. The modeling activity  in Fig. 4 means to create a model that conforms to the metamodel shown in this section.

### Modeling the Infrastructure

**Fig. 6:**
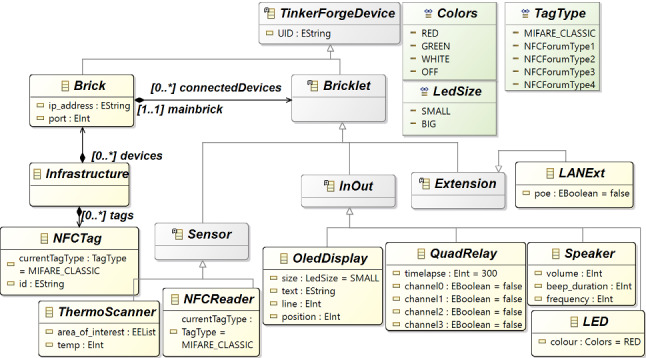
Tinkerforge Metamodel

To better explain the design of the chosen infrastructure and to enable the code generation, we engineered the Tinkerforge architectural modules in a metamodel, partially shown in Fig. 6. We omitted other modules we did not use in our case study to make the picture readable. An *Infrastructure* is composed of *Brick*s, to which an application can connect through its *ip_address*. *UID* identifies a building block. A Brick can be connected to *Bricklet*s, which can be classified into various types, for instance, *In/Out* devices, *Sensor*s and *Extension*s. The *NFC Reader* is of type *Sensor*, and it has to declare which *TagType*s it reads for the targeted application. Also, the *ThermoScanner* (i.e., the *ThermalCamera*) is a type of sensor, and among the attributes that can be specified there are the detected temperature and the spotmeter *area of interest*. The *Speaker* (beeper) can be set with specific beep duration, volume, and frequency. The *QuadRelay*, instead, requires the time-lapse for opening the channel set to true. *NFC tag*s contains a unique identifier and all the sectors to read and write. The structure of a tag is divided into sectors composed of blocks. However, since the application is simply reading the tag’s UID, we do not read and write the sectors (for this reason is omitted from the metamodel). Modeling the infrastructure following the rules and concepts in this metamodel means fulfilling the modeling activity  in Fig. 4.

### Modeling the behavior of the system

This section briefly explains the formalism used to express the ACS application’s behavior. Harel [46] defined the statecharts formalism, which can be used to simplify the specification of reactive systems [47]. It is a graphical notation that can be considered an enhancement of the finite-state machine (FSM) and their representation as state transition diagrams [48]. An FSM is composed of a set of states and a set of transitions. It delivers state transitions and produces outputs through input and by employing the current state. We used Yakindu Statechart Tools^3^, which is a statecharts modeling tool offered with an Eclipse pre-packed bundle. Unlike FSM, a statechart also allows modeling additional elements, such as composite states, events, variables, and actions. Thus, we decided to use Yakindu because it allows defining these elements to be used in the statechart and for the technical space completely integrated with EMF.

**Fig. 7:**
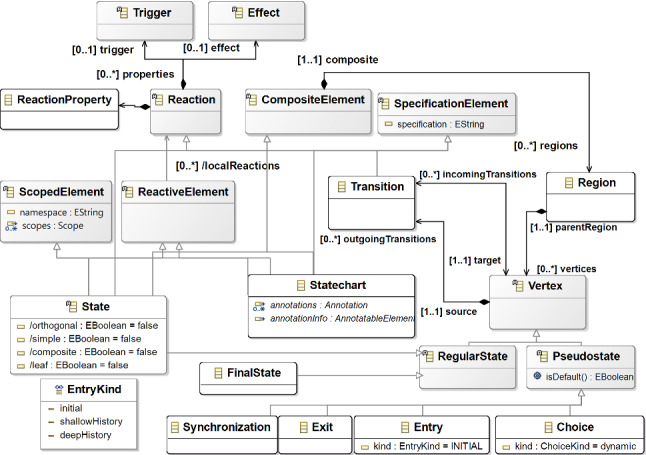
Yakindu statecharts Metamodel

The Yakindu editor permits to define a statechart model that conforms to a metamodel, reported in Fig. 7. The main element is *Statechart* metaclass, which contains one or more top-level *Region*s (*regions* composite association coming from *CompositeElement* super-metaclass) used to model statecharts. A region includes *Vertex*s that can be *RegularState* or *Pseudostate*. A regular state element is specified through the sub-metaclasses *FinalState* or *State*. A state can be simple or composite by specifying the *simple* or *composite* attributes, respectively. The Pseudostate metaclass allows defining conditional path (*Choice* metaclass), forks and joins (*Synchronization* metaclass), entry (*Entry* metaclass) and exit (*Exit* metaclass) pseudostate. Concerning the state transitions (*Transition* metaclass), each vertex can have several outgoing transitions (*outgoingTransitions* composite association), each of them connected to a target state (*target* association). Each transition is composed of a trigger (*Trigger* metaclass), a guard (*specification* attribute coming from *SpecificationElement* super-metaclass), and an effect (*Effect* metaclass). A trigger is an event that raises the transition if the guard condition is satisfied. When the transition is taken, its effect actions are executed.

When the infrastructure modeling activity  is completed, a minimal statechart model will be generated to start the behavior modeling activity , with a minimal configuration that we will see in the following sections. The arrow in Fig. 4 going from $\rightarrow$  using a first-code generator generates the interfaces and operations available for the configured infrastructure in . In this way, when modeling a state, the user can associate with the action to be triggered only the available operations from the infrastructure. This assures that the modeler cannot bind an action to a non-equipped bricklet. For example, she cannot map the state’s action to the led blinking if the LED bricklet is not equipped on the modeled brick. At this stage, the user can complete the statechart to represent the expected behavior of the brick.

### Modeling the interoperability

**Fig. 8:**
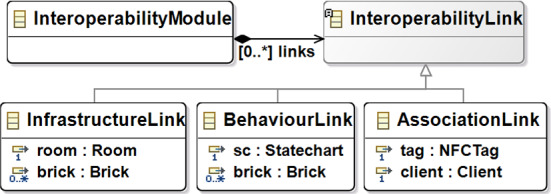
Interoperability Weaving Metamodel

At this point, we have a metamodeling layer supporting the definition of different types of models: the infrastructural model (conform to the metamodel in Fig. 6), the access control system model (conform to the metamodel in Fig. 5), and the statecharts (conform to the Yakindu metamodel in Fig. 7). At this stage, the missing information, as regards the approach shown in Fig. 3, is about how the infrastructure is related to the concepts we have in the ACS. For instance, how the room’s door is controlled by a specific brick and, in particular, an NFC reader. Moreover, we can also relate NFC tags to the clients to keep track of the authorized accesses, exactly as a web application managing human resources does. To do that, we conceived a weaving metamodel, namely *Interoperability Weaving metamodel*, which is shown in Fig. 8. A weaving model is a model specification used to capture relationships between model elements [49]. In this metamodel, we have three types of links: *InfrastructureLink*, defining how the building blocks of our infrastructure can be related to concepts of the ACS, e.g., room and brick; *AssociationLink* defining that a specific tag has been assigned to a client. Basically, the process starts from the definition of the models of the infrastructure (see Fig. 6) and the ACS (see Fig. 5) and enables the automatism summarized in Fig. 3. Moreover, as soon as we can model the system’s behavior using the statecharts, we still need to define how the statechart models the behavior of a specific brick. We can specify in a weaving model how our infrastructure should act in terms of behavior. Using this metamodel, it is possible to specify a set of *BehaviorLink* (the third type of interoperability link) establishing relations between a statechart model (defined with the formalism explained in Sect. 4.3) and a specific brick of our infrastructure (explained in Sect. 4.2).

The *Interoperability Weaving metamodel* can be enriched with other types of links, and it is still under development. With respect to Fig. 4, this weaving approach supports the activity  and enables the code generation  that will be discussed in the rest of this section.

### Modeling the ACS authorization mechanism

Our approach comes with an authorization mechanism that, by default, is designed to use model-based artifacts. This is what we identify in Fig. 3 with *ACS Auth script*. This component is formed by a Java method called isAuthorized() that we reported in Listing 2. Statechart states can be associated with this method to receive the authorization of a specific tag/user for a specific room (identified by the NFC UID). What we propose in the following is a script that queries the *ACS model* in order to get authorization. However, it can be replaced by a REST API call communicating with a Web platform, as we have seen in Sect. 3. The target platform includes this script, and we customized it by manually writing the code executing the authorization mechanism. This code stub returns true or false and can be customized by simply reporting the code responsible for calling the REST API and managing the authorization externally.
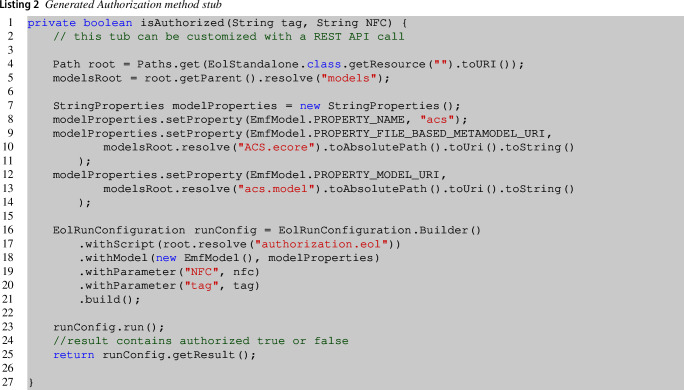
 This Java code executes an Epsilon Object Language (EOL) [50] query on the ACS model in a standalone mode. EOL is part of a family of languages implemented in Java for automating model manipulation tasks. EOL is a scripting language that combines the imperative style with the functional model querying capabilities of OCL. The code reported loads as parameter the NFC of the room in which the user is trying to access with a specific tag also passed as a parameter. The EOL script is loaded at line **17**, while the ACS model is loaded at line **13**. Line **25** returns the result of the query that is true in case of authorization granted or false if denied. Executing the EOL query on the ACS model will retrieve information about if the tag has been effectively assigned to someone and if the client is authorized by traversing the ACS model. The executed queries are executed by the PDP and are stored in a dedicated repository that the administrator can further extend with additional authorization mechanisms. Listing 3 reports an example of policy and corresponds to the authorization query that the API can trigger on an external third-party access-control client management platform. 
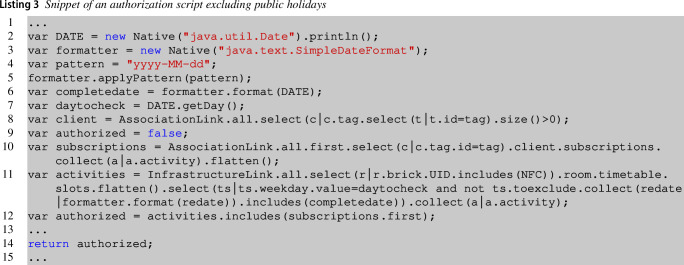
 This authorization policy shows additional constraints, excluding, for instance, public holidays specified by the administrator. This query introduces a specific check at line **11** by exploiting the model specification of the *TimeSlot*, in which the modeler can specify dates to be excluded from the schedule by using the attribute *toexclude* (see metamodel in Fig. 5). For instance, if Christmas falls on Friday, then the modeler can indicate the exclusion criteria of the time slot as a date. Line **11** additionally checks if the access date is defined as exclusion criteria and, in this case, returns an empty list of activities, i.e., user not authorized. We remind that further authorization policies can be provided by the policy administrator by simply adding a new EOL query to the repository and selecting the preferred one. Other examples can be found on our online material^4^.

### Code generator

An automatic code generation is a generic approach in which the same generator can produce different artifacts according to the inputs it receives. Template-based code generation (TBCG) emerged as an approach to develop code generators [26]. In particular, it requires less effort from the programmers, reduces the possibility of incurring errors, and favors code reuse [51]. TBCG is a widespread technique in MDE, and they both emphasize abstraction and automation.

Our approach automatically generates (the code generator is triggered in  in Fig. 4) the code interacting with the Tinkerforge APIs as (manually) defined in Sect. 3 [14]. It has been extended with respect to its first version in [14] in order to generate the logic of each brick, representing it with a statechart. We used the Acceleo framework^5^ to generate Java code for the case study, but it can be easily extended to support other target languages, e.g., PHP (as done, for instance, in [52]). Acceleo is a template-based technology to create custom code generators. It allows developers to generate code from high-level models without worrying about how to parse and traverse input models. Indeed, our code generator takes the *Interoperability Weaving Models* as input. It navigates to the left and right models to get the right model elements and generates the code interacting with the Tinkerforge API, exactly as shown in Sect. 3. What is more, the proposed approach generates code for the Spring ecosystem^6^ that is composed of: (i) Spring Boot and Apache Maven^7^ allows for the creation of the stand-alone Java application; (ii) Spring Statemachine^8^ makes it to define and use state machine concepts with Spring applications; (iii) Tinkerforge Java library provides support with devices.

For the sake of clarity, the ACS model could be used to generate part of the Web application managing the data, as well as the SQL script of the application [53]. It is out of the scope of this paper, and this part is also managed with a model-based representation. In this section, we show the code generator generating code supporting the structural variability of the models, i.e., code related to the brick, and the mounted bricklets for the modeled infrastructure, in Sect. 4.6.1. This code supports the operations that can be invoked by the behavioral aspects of the system. Instead, Sect. 4.6.2 will show how we can generate the flow of our access control application from statecharts models. This code regards the interaction between the mounted bricklets and uses the generated code starting from the infrastructure model.

#### Code generation for structural variability

This section shows the code generator composed of multiple templates addressing the structural variability. The first template used by the code generator is reported in listing 4. This template uses the three metamodels reported in the previous sections, loaded at line **2**. Indeed, it takes as input the Interoperability Weaving model, which also includes external resources, i.e., the ACS model and the Infrastructure model. This template called generateElement starts the generation from the declared InfrastructureLinks and, for each brick linked to a room, it generates a java class called BrickconfigurationXYZ.java, where XYZ identifies the UID of the brick (retrieved from the model). For every room in the ACS model, the generator will create a package (line **10**), including the java classes for all the bricks mounted for that room. In this way, the user can easily distinguish between the generated infrastructures. Lines **16–18** implement what we call selective import statements (see Table 1). The selective import statements are important, especially for micro-controllers, because they will have no impact on the program’s runtime speed, affecting the compile-time speed. Also, they improve the readability of the code and, consequently, the maintainability^9^. The selective import section is supported by the invoked template genSelectiveImport() (lines **38–46)** that processes the bricklet passed as a parameter, and in case the brick is equipped with a specific bricklet, it generates the import statement. Then the template continues by processing the variables declared in lines **30–33** in which the generator writes the UIDs variables for all the connected bricklets. Also, in this case, this iteration is obtained by navigating the infrastructure model. The latest template invoked in this generator is called genSelectiveBeans, in which all the code needed to initialize the bricklets equipping the brick will be generated. An example is reported in lines **50–69** where the beans^10^ for the NFCReader and for the QuadRelay are generated. This first template outlines that the structural variability is supported since the generated code refers only to the equipped bricklets on the various bricks. The developer will obtain, for each room, the structural initialization code needed.
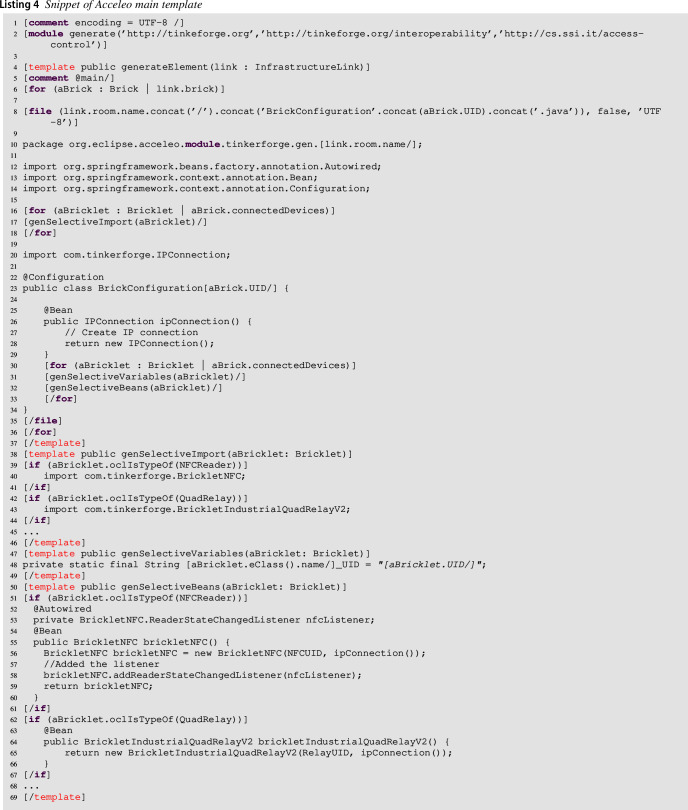


When this first-code generation phase is concluded, our approach automatically generates the code from the statecharts linked to the available bricks.

#### Code generation for behavioral variability

This section shows the template of the code generator supporting the behavioral variability of the approach. As already said in Sect. 4.3, the modeler shapes the statechart for each brick by starting from the partially generated one. In particular, each state uses the available operations generated from the infrastructure. Listing 5 is in charge of generating the available modeled states of the statechart as well as the statechart configuration, taking into account the available action mappings. This module begins with the template generateElement that generates the code from the Interoperability Weaving model. Then, the template selects the BehaviorLinks (lines **4–10**), and from the related Statechart to each declared brick (lines **5–9**), it generates an enumeration containing all the states declared in the statechart (line **6**), the related state actions (line **7**), and state machine configuration (line **8**). The detailed code corresponding to lines **6–8** can be found on our online material^11^.
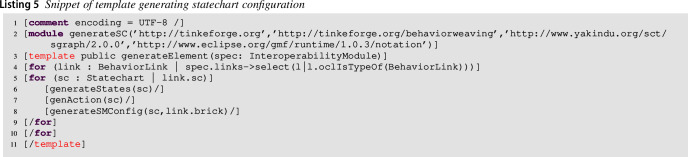


### Framing our approach as a model-driven security approach

Model-Driven Security (MDS) has emerged as a specialized Model-Driven Engineering approach for supporting the development of security-critical systems [2, 3]. In order to classify our approach in the MDS domain, we evaluate it against the taxonomy defined by Levi et al. [2] reporting about the essential concepts of the MDS domain. Table 2 lists a set of concepts relevant to MDS, with their brief description about how these concepts are realized in our approach.

**Table 2:** A taxonomy for model-driven security approaches [2]

Taxonomy Entry	Description in our approach
Application domains	*Is the approach domain specific or generalistic?* Our approach is **domain-specific** in the access security context.
Security concerns	*Which concerns does the approach focus on?* **Authorization** (i.e., ACS) and **authentication** (i.e., NFC tags).
	*Are they expressible in a metamodel?* Yes, e.g., the Access Control System Metamodel (see Fig. 5).
Modeling approach	*Which Modeling paradigm(s)? Which Modeling language(s)?* Our approach makes use of a defined **domain-specific language**.
Separation of concerns	*Is it used? If it is, how is it implemented?* SoC is realized through **policies** for the authorization logic and **weaving modeling** for the ACS and the infrastructure (see Fig. 3).
Model transformations	*Are Model-to-Model/Model-to-Text transformation used?* **Model-to-Text** transformations are used for statecharts and devices code.
	*Which model transformation engine is used?* Acceleo is used as transformation engine.
Verification	*Which verification techniques are used?* No formal verification approach is explicitly used.
Traceability	*Are backward and/or forward traceability implemented?* Backward and forward traceability offered by Acceleo is exploited.
Tool support	*What is the automation level of the approach?* Basic UI relying of pre-configured technologies.
	*Which features does it provide?* Default modeling editors: EMF; Fully automated code generation: Acceleo;
	Automatic deployment supported by the target platform: TinkerForge.
Validation	*Is the approach validated on large, meaningful cases? Has it been* *industrially validated?*
	Our approach has been applied on a **real-world case study**, i.e., a fitness center (see Sect. 5).

In summary, our approach represents a domain-specific approach in the access security context, managing both the authorization and authentication security concerns by implementing an ACS. Our approach uses a defined DSL that advocates a clear separation of concerns through policies for managing the authorization logic and weaving modeling for the ACS and the system’s infrastructure (see Fig. 3). The bridge between the used models and the final code is supported by model-to-text transformation features through the Acceleo engine. Moreover, due to the traceability between the generated code and corresponding models offered by Acceleo, and given that we use weaving models, we can say that our approach provides both backward and forward traceability. More precisely, this is true as long as we can identify that errors depend on the generator or issues derived by model compositions. As regards verification, although our approach does not make use of formal verification approaches, the statistics obtained from collected data provided by the real-world case study (see Sect. 5) show that users have raised no issues. In other words, although statistics about the usage of the generated code are not exhaustive, they would prove that the generated code acted as expected. Lastly, our approach shows a basic UI relying on pre-configured technologies, as summarized in Table 2. However, the tool can be further automated and extended. As future work, we aim to provide a fully integrated platform, including features for validation and simulation, not yet supported. The validation through a real-world case study proves the usefulness and correctness of the approach. Even if we use a fully model-based approach as a proof-of-concept, the realized ACS has been deployed as a hybrid solution, as described in the following section.

Eventually, in their work, Nguyen et al. [3] complain about “*the lack of all-round approaches for the whole development cycle of secure systems which in the end lead to automatic generation of both code and security infrastructure.*” We believe that our approach basically realizes a *all-round approach* for the whole development cycle of secure systems.

## Our approach at work

This section describes our approach at work on the use case described in Sect. 3. As already said in Sect. 4, we have to specify the infrastructure model, the ACS model, the relationship between infrastructure and ACS models (i.e., the weaving model), and the statecharts related to each brick, respectively. Then, the code generators produce the Tinkerforge code to be deployed to the real system.

**Fig. 9:**
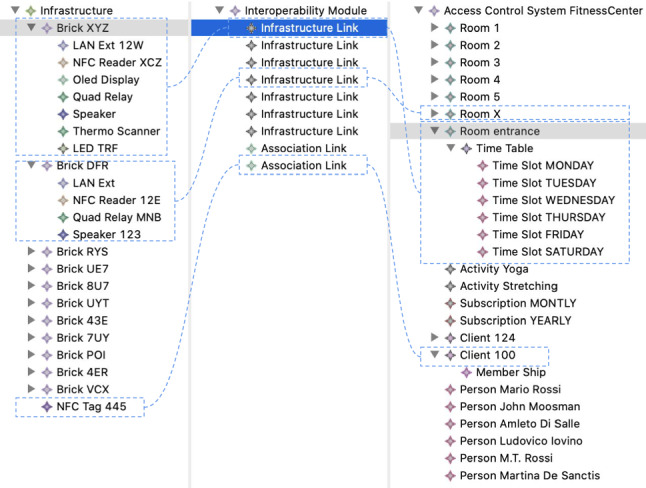
Interoperability weaving example model

Figure 9 reports a screenshot of the weaving editor, where an interoperability weaving model (modeled in ) has been created to link the infrastructure model (modeled in ) with the ACS model (modeled in ). The dotted lines represent the links between the concepts. For instance, the brick with UID XYZ has been linked to Room Entrance. Every room has been set up with the corresponding activities and time slots. Moreover, we have some associated tags, e.g., the tag with id 445 with client 124, aka Mario Rossi. Not all the properties of the model elements are visible in this screenshot, where we tried to highlight the links defined in the weaving model. What is important to notice here is the *Infrastructure Link*s in the interoperability model embodying the information about which brick is mounted in a specific room. For instance, the first brick with UID XYZ is mounted at the Room Entrance, as can be seen from the relation that the weaving tool automatically highlights. Furthermore, the bricklets mounted on top of the brick are listed in the selection. The same applies to the brick with UID DFR mounted on Room X. It is evident that the mounted bricklets effectively shape different configurations from one room to another.

**Fig. 10:**
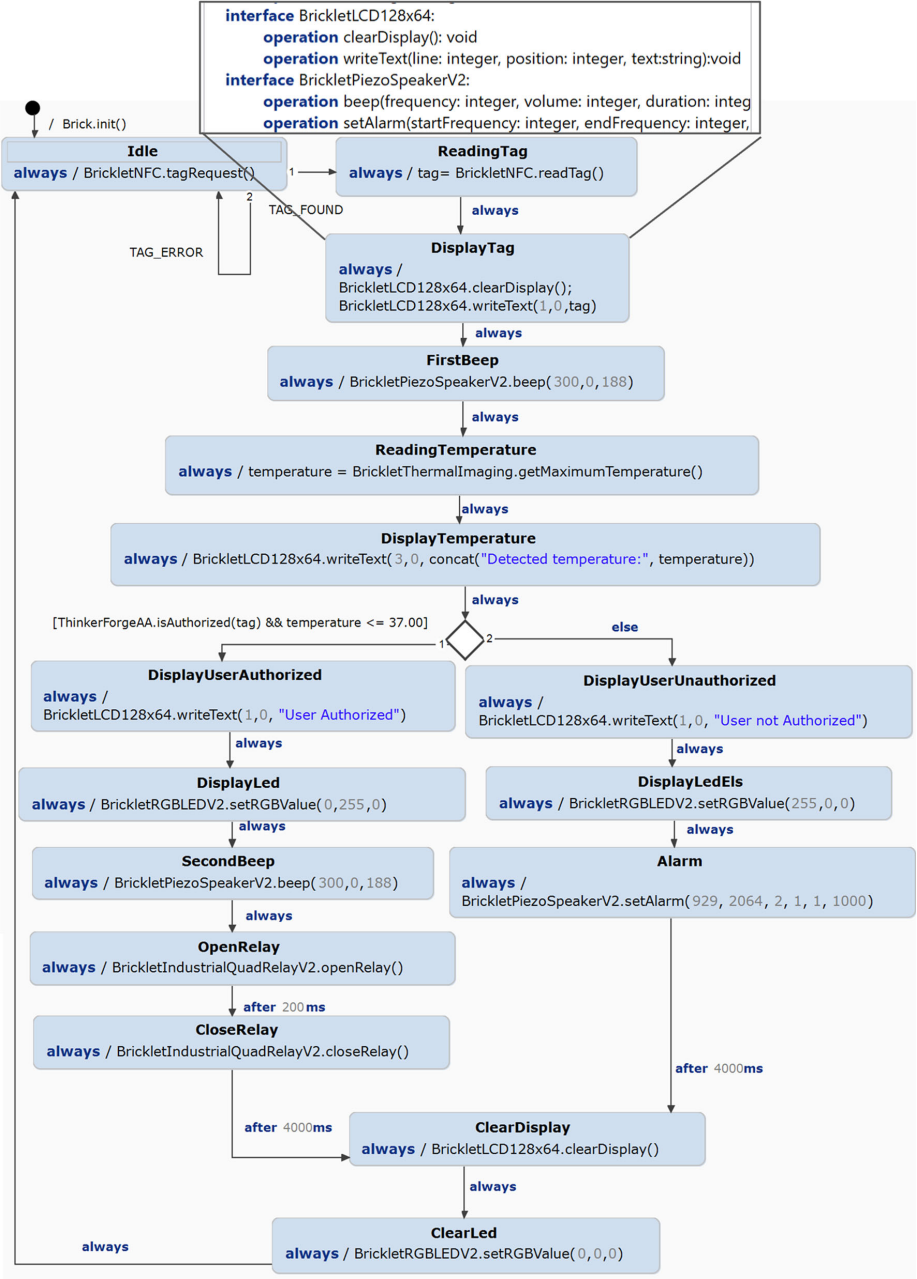
Entrance Brick statechart

Figure 10 shows the statechart used to model the *entrance* brick behavior in  and that we used to generate the code running and interacting with the device^12^. On top, we reported a snippet of the interface with the operations generated for each bricklet from the infrastructure model. In particular, for each bricklet of the infrastructure, an interface with the available operations is generated. For instance, if the LCD display is mounted, for the DisplayTag state, an interface and two available operations (clearDisplay and writeText) are generated. This first automation is important since it generates only the available operations for the equipped bricklets. Since our approach is based on NFC bricklet, the Idle and ReadingTag states are automatically generated. Together with their transitions, they model the request and reading of a tag^13^.

The statechart models the same actions flow and events described in Sect. 3 in Listing 1. Moreover, we modeled the other three statecharts to cover the three possible configurations we described in Sect. 3 for the diverse room types.

**Fig. 11:**
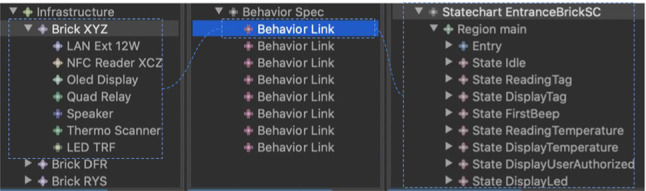
Weaving model used link modeled statecharts with the infrastructure

In order to express the behavior of each brick, we used the metamodel in Fig. 8 to model the correspondences as reported in Fig. 11. In this excerpt of the weaving model, we can see that the highlighted behavioral link establishes which statechart has been selected for a specific brick. For instance, in this case, the statechart reported in Fig. 10 has been linked to the entrance brick, identified with the UID XYZ.

After the modeling activities are finished with , the code generation phase takes place as indicated in . The code for structure variability is generated from the infrastructure and ACS models with the related weaving model. The behavior code comes from the statechart models for each brick, together with the weaving model.

Listing 6 is an excerpt of the code generated for the brick configuration related to the entrance brick. In particular, lines **1–6** define a bean for the NFC bricklet, while lines **7–10** specify a bean for the piezo-speaker bricklet.

Listing 7 shows an excerpt from Brick statechart generated code again for the entrance brick. The template generates all statechart states (lines **2–7**), and the statechart transitions between states (lines **9–16**). Lines **18-27** specify the action related to the DisplayTag state.

By executing the generated code for the complete infrastructure and deploying the generated code on the devices or a centralized server, we managed to test the communication mechanism with the infrastructure we modeled.

**Fig. 12:**
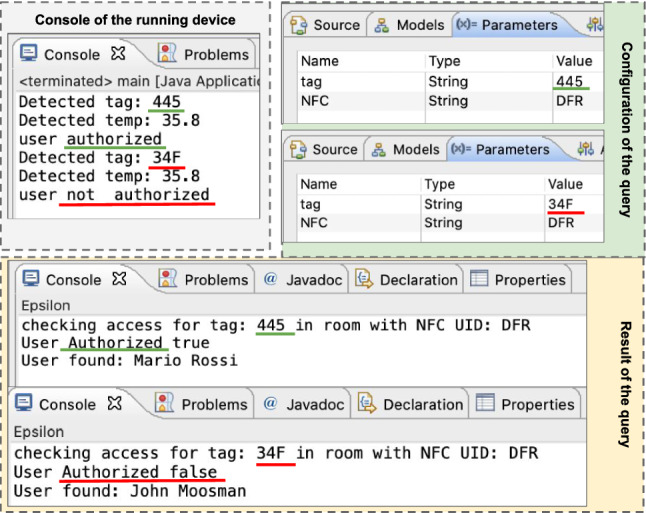
Console log showing the runtime interaction of one of the access control devices in the fitness center premises

The log in Fig. 12 (top left) reports the printed detected tag id by the device running the generated code, e.g., 445 (authorized) and 34F (unauthorized), corresponding to specific clients interacting with the ACS. The bottom part of Fig. 12, indeed, reports the result of the authorization script (reported in Sect. 4.5) executed crossing the detected tag by the NFC reader with UID DFR, with the clients and activities of the room infrastructured with that brick. In Fig. 12 top-right, we reported the configuration of the EOL query on the model, which corresponds to setting the two parameters passed to the authorization script called in Listing 2, i.e., the tag id and NFC UID.

We can conclude that the two used tags resulted once in the authorized and once in the unauthorized access.

Real Case Study Stats Considering that the code generated for the ACS is actually in use and running in a fitness center, we report some stats obtained from the collected data. Table 3 reports the summary of the entries authorized or not for all the rooms in the building in a time window of 2 months. All the bricks run with generated code and, specifically, for each device of each room depicted in Fig. 1, two rows are reported in Table 3, showing the number of authorized () and denied () entries, respectively. We recall here that some rooms have two entries, each with its device (e.g., entrance.1 with the device JKV, entrance.2 with the device JL2), while others have only one entry equipped with one device (e.g., room 1 with the device JL9). Moreover, the authorization script of the PDP has been replaced with the REST API call since having more than 2000 clients accessing the model managing the tag association would result in rapid degradation of the performance w.r.t. a REST API call.

If we focus on the entrance, we can easily see that the two devices in this room managed a total of 10872 entries over two months, of which 469 have been denied, while 10403 have been authorized. This further shows how the ACS supports the fitness center’s management by efficiently detecting expired subscriptions attempts to participate in activities that are not part of the granted subscription or simply entries attempts by unauthorized people. Even if our work focuses not on generated code correctness, it depends on the models and the correctness of the code generator templates. Therefore, we outline that the generation has effectively distributed the generated code on a real case study already running.

## Evaluation

This section reports the evaluation of our approach in terms of *automation* with respect to traditional code-centric techniques and *time to market* (TTM). These represent particularly relevant aspects in the case of a code generation approach. Thus, we propose to evaluate the following research questions:

- *RQ1* How well can the presented modeling approach reduce the effort of a developer in terms of lines of code?
- *RQ2* How well can the presented modeling approach reduce the effort of a developer in terms of time to market?

In this evaluation, we compare the traditional code-centric development of the case study shown in Sect. 3 with the presented modeling approach.

### Experiment setup

In order to answer the defined research questions, given the described case study, we compare the lines of code that a developer should write to implement it with respect to composing the model defining the case study (identified as modeling activities $\rightarrow$ $\rightarrow$ $\rightarrow$  eventually triggering ). We have selected two persons with different backgrounds and profiles: one is a modeling expert (called *modeler* in the remaining of the section) with a postdoctoral fellowship position, not aware of the presented modeling approach, but with a strong background in MDE and the Eclipse Modeling Platform. The other is a student of the master in mobile and web technologies^14^ (called *developer* in the remaining of the section) with expertise in Java EE application development and web development in general.
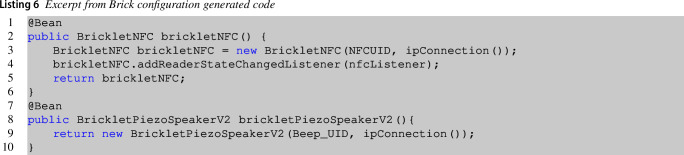

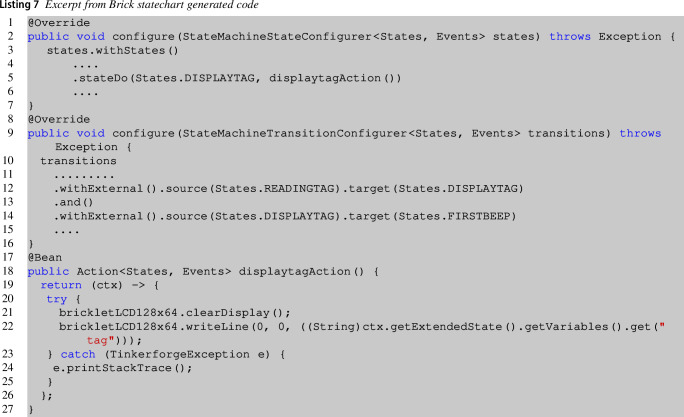


A training phase anticipated the conducted experiment in which we only showed the Tinkerforge general architecture and the architecture of our generated approach by referring to the entrance brick (the most complete) (2 hours in total). Moreover, a general description of the client flow has been shown with an example of the brick usage. Then we referred to the Tinkerforge documentation website. The modeler has been trained with the same process, and additionally, we have shown examples of the modeling artifacts we have developed for testing (as Fig. 11 for instance). The developer had the Tinkerforge documentation available where examples are shown, so we think the two training phases correspond. After the development/code generation phase, we tested the source code in the simulation environment with the entrance brick connected to our LAN. We marked the code as correct if the minimal flow described before was respected.

The used metric, i.e., *Lines of code* (LoC) [54], is a widely used metric to measure the size of a software program by counting the number of lines in the text of the program’s source code [55]. This metric is typically used to predict the amount of effort required to develop or maintain software. For this reason, we used it to estimate the developer’s effort. On the other side, in our approach, the modeler has to compose the interoperability module models to produce the source code. For this reason, we rely on the *Model Size* metric [56] that is simply counting the number of instances that have to be created to compose the model, e.g., number of *Infrastructure Link* or *Association Link*, *Brick*s or *Room*s. Moreover, the attributes and structural features set in the model contribute to the model size as in the following formula:

1 $$\begin{aligned} \small {Size(Model) = nr\_instances + (0.5 * nr\_set\_features)}\nonumber \\ \end{aligned}$$

The differences in terms of LoC between the manually written application and the generated one were minimal, and we did not compare the code since it is out of our experiment. This formula has been embedded in an EOL script that we do not report since it is quite trivial. It calculates the size of the model passed as a parameter by counting its instances, and, for each instance, it counts the number of the filled attributes and references. This comparison is used to answer to **RQ1**. Differently, for **RQ2** we make use of another comparison. When using a model-based approach, the effort can be measured by how much time a modeler needs to compose the model. This composition activity comprehends the editing of the model by alternating the addition of new instances and filling in the required information in the property view. In this case, the modeler composed the weaving model containing all the configured bricks and generated the entire code. Thus, we could estimate the LoC for the other bricks.

**Table 3:** Report on the building entries in a time window of 2 months

Room id	NFC	Authorized	Entries
entrance.1	JKV		312
entrance.1	JKV		8200
entrance.2	JL2		157
entrance.2	JL2		2203
2.1	JKW		139
2.1	JKW		5098
2.2	JL6		96
2.2	JL6		4491
X.1	JKZ		18
X.1	JKZ		237
X.2	KwP		7
X.2	KwP		128
3.1	JL7		274
3.1	JL7		1875
3.2	Kx2		27
3.2	Kx2		461
1	JL9		86
1	JL9		649
4	JKY		243
4	JKY		1005
5	Kx1		97
5	Kx1		957

### Results analysis

The results of the evaluation for **RQ1** and **RQ2** are reported in Tables 4 and  5, respectively. From Table 4 it is evident that for each brick added to the infrastructure, the code to be developed increases in terms of LoC, whereas the model to be composed increases of a few elements. Consider that the average model slice [57] size for adding the complete management for a single room corresponds to 50, whereas the average LoC for a single room ranges between 200 and 219 in our experiments. In fact, the lines of code developed for the case study in Sect. 3 are 1459. The model to be composed has a size of 248 elements, including three versions of the statecharts for three different configurations.

**Table 4:** Evaluation results for RQ1

Configuration	LoC	Nr. Bricks	Tot. LoC	Model Size
Entrance	219	1	219	248
Room 1–5	208	5	1040	
Room X	200	1	200	
Total			1459	

Table 5 shows that the time spent to develop the Java code corresponds to around 12 hours. In particular, rooms 1–5 show different configurations, but with a slight variability, we start from a time of 2 hours for room 1, and then we decrease the time required for each room by 20 minutes. It means that the time required for rooms 1–5 ranges from 2 hours for room 1 to 40 minutes for room 5. On the opposite, building a model for the case study took 42 minutes from scratch, including the statecharts that are, in part, “conceptually” similar. We omit the code generation task since it takes only seconds.

**Table 5:** Evaluation results for RQ2

Configuration	TTM	Nr. Bricks	Tot. TTM	TTM
	Coding		Coding	Modeling
Entrance	4 h	1	4 h	42 m
Room 1–5	2 h 40 m	5	6 h 40 m	
Room X	1 h	1	1 h	
Total			11 h 40 m	

The evaluation result shows a drastic reduction in terms of manual activities and time spent to get the code running. We also verified from this experience that the more the gates of the building increase, the more the variability grows. This also supports the usefulness of this approach, especially for buildings with a large number of rooms to be equipped.

### Threats to validity

According to Wohlin et al. [58], the validity of a study denotes the trustworthiness of the results and to what extent the results might be biased by some factors, e.g., the researchers’ subjective point of view. In the following, we discuss the four classical aspects of validity, namely construct, internal, external, and conclusion validity, plus reliability.

Construct validity It refers to the appropriateness of measurements applied to evaluate the approach. To minimize the threat to construct validity, we defined all the details regarding the performed evaluation a priori. Before proceeding to the realization of the case study, the authors agreed on the research questions to be evaluated, the evaluation methodology to be followed, and the metrics to be exploited. It allowed us to discuss every evaluation aspect and avoid different interpretations at evaluation time.

Internal validity It refers to the presence of potential biases that might affect the evaluation results by making them questionable [58]. The presented approach is based on the Tinkerforge platform. Currently, the actual implementation of the specified metamodel does not comprehend all the possible bricklets that Tinkerforge provides. The code generation process has been tested only for the devices we currently have by making the Tinkerforge platform underused. However, the approach we proposed is general, and it can be further extended to support the complete set of bricklets.

A second internal threat validity is that the results have been obtained on a case study made by a limited number of bricks. Indeed, we defined a real setting by modeling/instantiating all the bricks required from the given fitness center. We are aware that this limited number could threaten the internal validity of our experiment. To strengthen our study findings, we should consider further case studies requiring a more significant number of bricks in broader experimentation.

A third internal threat validity refers to the complexity of modeling, which is not captured by the model’s size metric and cannot be easily compared with the manual code effort. However, the modeling strongly supports abstraction, reuse, and code generation, at the expense of the developers’ modeling effort. We believe that adding modeling effort is a good compromise for writing the entire system code, thus overcoming the limitations of code-centric development, e.g., proneness to error. Moreover, as argued in the external validity paragraph, the modeling features also help tackle cross-platform development.

Eventually, we are aware that the experiment should involve multiple persons to avoid a learning effect if the same system is developed using both approaches. Since we did not have a team of modelers and developers involved in the experiment, and this task was very time-consuming (on one side), we mitigated this threat by distinguishing the tasks of each user; one user has been employed in the code development and the other one in the modeling approach. The last point we need to consider is that the experiment was conducted remotely and in asynchronous mode, meaning that we had a conversation with the involved users and were only asked to provide the TTM and LoC when the assigned task was complete. The provided TTM could be a rounding of the adequate time spent developing the code, e.g., 58 minutes could be rounded to 1 hour. The difference between the TTM with coding and modeling is so reduced. Thus, we can state that the approach does not suffer much from this threat. This can only be the case in which the case study is based on a single brick with a minimal number of equipped bricklets.

Reliability We compared our modeling approach with respect to traditional code-centric approaches using two different metrics, i.e., LoC and the models’ size. Regarding the reliability of the measures used in our evaluation, we are aware that this comparison is limited by the difference between the metrics used, not being directly comparable. However, as stated by Wohlin et al. [58], objective measures are more reliable than subjective ones. In this regard, lines of code and the models’ size are considered more reliable metrics since they do not involve human judgment.

External validity It concerns to what extent it is possible to generalize the findings [58]. For generalization, we discuss the following points. The approach has been used to develop an access control system. Consequently, the presented statecharts are currently limited to the representation of the behaviors within the realized system. Different systems might imply the need for more complex statecharts, which could threaten our approach’s external validity.

Another threat to the external validity is that we currently generate only the most common operations used by the bricklets and not all the possible operations that bricklets can perform from the presented statecharts. For instance, for the speaker bricklet we support the beep operation through parametric frequencies. However, the speaker also supports the management of given alarms that we do not handle. Anyhow, the set of generated operations, despite not being complete, covers all the required functionalities for realizing the necessary behavior of an access control system.

Our approach has been explicitly realized to be used with NFC technology. For this reason, we assume that bricks are always equipped with an NFC reader and clients with their tags. This assumption is reflected in the code generation templates and, in turn, in some of the generated code lines. For instance, the used statecharts, such as that reported in Fig. 10, always include the *readingTag* state. Although it may seem that this assumption limits the level of abstraction of the approach, we believe that it facilitates both the modeling and the code generation of the ACS in cases where the application is intended to be used with a specific reference technology, such as NFC, in our case.

The presented approach is generalizable and applicable for developing different versions of ACSs that make use of the Tinkerforge sensors technology. Indeed, the approach supports modification to the system’s architecture or to the infrastructure and its building blocks. This is also due to the template-based code generation offered by the approach. In fact, although writing templates is not an easy task with respect to directly writing the system code, it provides relevant support for reuse.

Threats to external validity can arise only in two cases, i.e., when a change of programming language or a platform change is required. As explained, Tinkerforge supports many programming languages, e.g., Java, PHP, and Python. Moving from Java to a different language would require a new code generator for the new supported language without changing the used models. Passing from Tinkerforge to a different target platform would imply changes in both the templates for the code generation and the used models, at least those closely related to Tinkerforge.

Conclusion validity It regards issues affecting the ability to draw the correct conclusion of an experiment. To mitigate this threat and avoid individual bias and interpretation of the results from the side of the authors, we involved a modeler and a developer in the evaluation of the case study. This helped us to draw our conclusions more objectively.

## Conclusions and future work

This paper presented a model-driven approach, supporting automatic code generation to enable communication between an IoT infrastructure and ACSs. This work has been triggered by the need to migrate an old chip card-based access control system to a new way of authorizing people with NFC tags. In particular, the approach enables the generation of software components in the selected target language (e.g., Java) by also offering a model-based authorization mechanism that can be easily replaced by a third-party system REST API call. The objectives of the presented approach are twofold: (i) relieving developers from the inherent complexities of heterogeneous IoT devices, protocols, and networks, also in view of a multi-platform evolution of the approach; (ii) making the whole development process less error-prone and time-consuming, while exploiting code generation.

As future work, we plan to address the limitations discussed in Sect. 6.3 and to make the generative approach multi-platform by targeting additional languages besides Java through broader exploitation of the Tinkerforge platform. Another intriguing clue is to provide the authorization mechanism in a model-based representation of the policy [59], which is currently managed by a repository of authorization scripts. In the case of changing the access policy, only the security-level changes by redeploying only the new policy and its decision point. In the future, it could be considered to customize the approach further, for instance, by enriching the weaving modeling phase with domain-specific graphical dedicated editors to be used instead of the one based on the panels. Low-code platforms seem to be a natural choice for multi-platform integrated graphical modeling. Moreover, it would be interesting to experiment with multiple architectural deployment alternatives, e.g., centralized, decentralized, and microservice-based, to check the versatility of the generative approach and how much effort is required to adapt it.

## Footnotes

1 The case study was carried out by us on a non-profit basis with the only aim of assessing our approach.

2 https://www.eclipse.org/modeling/emf/

3 https://www.itemis.com/en/yakindu/state-machine/

4 https://bit.ly/3j8pU3E

5 https://www.eclipse.org/acceleo/

6 https://spring.io/

7 https://maven.apache.org/

8 https://spring.io/projects/spring-statemachine

9 http://www.javaperformancetuning.com/news/qotm031.shtml

10 Spring framework defines a bean as an object instantiated and assembled by a Spring Inversion of Control container.

11 https://bit.ly/3j8pU3E

12 We left out of this model the modeling of the checks analyzing if the triggered action corresponds to an entry or an exit which is quite similar but interacts with the authentication script to write the exit date on the model.

13 https://www.tinkerforge.com/en/doc/Hardware/Bricklets/NFC.html

14 https://mwt.disim.univaq.it
